# Nondestructive Detection of Soluble Solids Content in Apples Based on Multi-Attention Convolutional Neural Network and Hyperspectral Imaging Technology

**DOI:** 10.3390/foods14223832

**Published:** 2025-11-09

**Authors:** Yan Tian, Jun Sun, Xin Zhou, Sunli Cong, Chunxia Dai, Lei Shi

**Affiliations:** 1 School of Electrical and Information Engineering, Jiangsu University, Zhenjiang 212013, China; ty@just.edu.cn (Y.T.); zhouxin_21@ujs.edu.cn (X.Z.); 2112407120@stmail.ujs.edu.cn (S.C.); txdcx@126.com (C.D.); 2112307128@stmail.ujs.edu.cn (L.S.); 2 School of Automation, Jiangsu University of Science and Technology, Zhenjiang 212008, China

**Keywords:** soluble solid content, apple, convolutional neural network, multi-attention, hyperspectral imaging technology

## Abstract

Soluble solids content is the most important attribute related to the quality and price of apples. The objective of this study was to detect the soluble solids content (SSC) in ‘Fuji’ apples using hyperspectral imaging combined with a deep learning algorithm. The hyperspectral images of 570 apple samples were obtained and the whole region of apple sample hyperspectral data was collected and preprocessed. In addition, a method involving multi-attention convolutional neural network (MA-CNN) is proposed, which extracts spectral and spatial features from hyperspectral images by embedding channel attention (CA) and spatial attention (SA) modules in a convolutional neural network. The CA and SA modules help the network adaptively focus on important spectral–spatial features while reducing the interference of redundant information. Additionally, the Bayesian optimization algorithm (BOA) is used for model hyperparameter optimization. A comprehensive evaluation is conducted by comparing the proposed model with CA-CNN models, SA-CNN, and the current mainstream models. Furthermore, the best prediction performances for detecting SSC in apple samples were obtained from the MA-CNN model, with an $R_{p}^{2}$ value of 0.9602 and an RMSEP value of 0.0612 °Brix. The results of this study indicated that the MA-CNN algorithm combined with hyperspectral imaging technology can be used as an effective method for rapid detection of apple quality parameters.

## 1. Introduction

Apple is a popular fruit favored by consumers for its nutritional value and health benefits. It contains various sugars, fruit acids, vitamins, cellulose, microelements, and antioxidant components, which can effectively reduce the damage caused by free radicals in the human body [1,2,3]. Soluble solids content (SSC), as an important inherent quality indicator of apples, is closely related to the taste of the apple and also affects the purchasing decisions and satisfaction of consumers [4]. Traditional SSC detection methods require the use of destructive digital refractometers, which are time-consuming and unable to quickly and efficiently determine the composition of the fruit flesh. Therefore, it is particularly important to develop a rapid, nondestructive, and safe detection technology to evaluate the SSC of apples [5].

With advancements in detection technology, various spectral techniques have been increasingly applied in the field of food analysis and detection, including visible and near-infrared spectroscopy [6,7,8], near-infrared spectroscopy [9,10,11], and Raman spectroscopy [12,13,14]. However, due to the uneven spatial distribution of chemical components within the sample, the information obtained through these spectroscopic techniques using point-source sampling is not fully representative [15]. Hyperspectral imaging (HSI) technology integrates the spectral and spatial information, which can detect the properties of samples nondestructively [16,17,18]. In previous studies, HSI technology has been successfully applied to quantitative and qualitative analysis of fruits such as apples [19], blueberries [20], pears [21], and grapes [22]. However, the spectral and spatial information of hyperspectral images are highly coupled, the increase in data dimensionality often leads to important features located within local band ranges being overwhelmed by redundant information.

Convolutional neural networks (CNNs) can learn deep abstract features from raw HSI data, avoiding complex expert knowledge and excessive manual intervention in feature extraction [23,24]. However, CNN processes data information from different spatial positions and spectral bands equally during feature extraction, making it difficult to extract higher quality spatial–spectral features. Meanwhile, processing all spectral bands at once to extract globally representative features makes the data susceptible to interference from redundant information, which limits the improvement of model prediction accuracy [25].

The attention mechanism can enhance crucial features while suppressing redundant information through weight mapping. Zhao et al. proposed a spatial–spectral transformation network that captures long-range spectral/image relationships via a multi-head attention mechanism for strawberry defect detection [26]. Roy et al. proposed an attention-based adaptive spectral–spatial kernel ResNet, which incorporates an adaptive spectral–spatial kernel and attention mechanism into the designed residual structure network. By dynamically adjusting the adaptive kernel based on the similarity and importance of samples, the model accuracy is significantly improved [27]. The mentioned studies demonstrate that employing the attention mechanism can effectively optimize the spectral–spatial feature extraction process, significantly enhancing model accuracy and robustness. Moreover, the reasonable selection of hyperparameters is crucial in deep learning modeling. Excessively high or low values of these parameters can lead to overfitting or underfitting of the model. Several popular hyperparameter optimization algorithms including grid search algorithms, particle swarm optimization algorithms, and Bayesian optimization algorithms (BOAs). It is worth noting that grid search algorithms require a large number of repeated experiments, which are time-consuming; particle swarm optimization is prone to getting trapped in local optima and exhibits low convergence accuracy. In contrast, BOAs obtain optimal parameters by introducing an acquisition function to evaluate the next optimization point [28].

Therefore, this study proposes a method that combines multi-attention mechanisms with CNN (MA-CNN) networks to extract spectral and spatial features of hyperspectral images using channel attention and spatial attention, respectively. By weighing the attention mechanism to enhance key features and weaken redundant information, the BOA is used to obtain optimized hyperparameters for the MA-CNN. Ultimately, a prediction model based on deep information fusion of spectral and spatial features is constructed to achieve nondestructive detection of apple quality.

## 2. Materials and Method

### 2.1. Apple Samples

Mature ‘Fuji’ apples were harvested from orchards in Qixia County, Shandong Province, China. All apple trees in the orchard were covered with two layers of paper bags two months after the peak flowering period. To ensure that the samples covered a wide range of growing conditions, apples were selected from different tree crowns and trunks, and three batches of experimental samples were collected, with each batch containing approximately 190 apples; a total of 570 apple samples with no obvious mechanical damage or defects were selected and transported to the laboratory of Jiangsu University, which had a temperature of 20 °C and a humidity level of 60%. The apple samples were placed in the laboratory for 24 h before the test to ensure that their temperature was consistent with the laboratory environment temperature. After that, all samples were cleaned and numbered, and the labeled samples were used for hyperspectral imaging system (HSI) acquisition and SSC value determination. In this experiment, the samples were divided into two groups at a ratio of 5:1, with 475 samples in the calibration set and 95 samples in the prediction set. And five-fold cross-validation was used in the calibration set to obtain the optimal network hyperparameters and to prevent the model from overfitting, through its combined use with the BOA.

### 2.2. Region of Interest Extraction and Hyperspectral Data Processing

**Figure 1:**
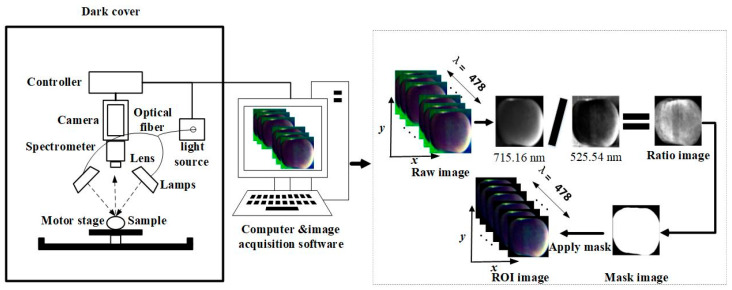
Hyperspectral imaging system and ROI extraction.

The hyperspectral imaging system with a spectral range of 400.648–1001.61 nm (with 478 bands) is presented in Figure 1. The system mainly included a hyperspectral imaging camera (ImSpectorV10, Spectral Imaging Ltd., Oulu, Finland), two optical fiber halogen lamps (3900-ER, Illumination Technology, Inc., New York, NY, USA), a CCD camera (Zyla4.2 Plus, Andor Technology, Inc., Belfast, UK), a mobile platform controller (TS200AB, Zolix, Corp, Beijing, China), and a computer. The spectral resolution was 2.8 nm, and the spatial resolution was 2048 pixels. Prior to obtaining hyperspectral imaging of the apple samples, the instrument was warmed up for half an hour. Subsequently, the distance between the upper surface of the apple sample and the CCD camera was set to 0.45 m. The CCD exposure times for the whiteboard, blackboard, and sample were set to 10 ms, 10 ms, and 17 ms, respectively. The speed of the sample stage movement was set to 3.76 mm/s, and the CCD camera and hyperspectral imager were each set to a 2048 pixels × 478 bands (spatial × spectral) mode for sampling the test samples. Finally, apple samples were placed in the displacement platform of HSI system, and hyperspectral images of each apple sample were then obtained one by one. In order to eliminate the effects of uneven illumination and dark current noise, the raw hyperspectral image was calibrated according to the following formula [29].

(1) $$I_{cal}=\frac{I_{raw}-I_{black}}{I_{white}-I_{black}}$$

where *I_cal_* is the corrected reflectance image, *I_raw_* is the original hyperspectral image, *I_black_* is the black reference image (with approximately 0% reflectance) obtained by covering the lens completely with an opaque black cover, and *I_white_* is the white reference image obtained by scanning a white standard plate with uniform and high reflectance (approximately 99.9% reflectance).

Extracting the region of interest (ROI) for the samples is critical in guaranteeing the reliability and typicality of the spectral information. Figure 1 shows the process of ROI extraction. Firstly, the spectra of samples were compared with the background region to obtain the bands exhibiting large and small differences (715.16 nm and 525.54 nm). The ratio image was obtained by converting the ratio between them. Then, a mask was obtained by using a minimum threshold of 1.6, which was manually selected according to the boundary critical value, with a large difference in reflectance between the target area of the apple sample and the interference background area. Finally, the original image was masked to obtain the target image. The average spectrum of the ROI was calculated to obtain data for subsequent modeling and analysis.

After data acquisition is completed, data augmentation is performed via data rotation and mirroring. In the hyperspectral image dataset, minor angle variations (randomly generating 3 rotations within different angular ranges, such as 0° to 30°, 150° to 180°, and 180° to 210°) are used, which enables the model to better adapt to various actual tilt angles. Subsequently, the image data undergoes mirroring in both left–right directions, which can increase data diversity. Specifically, a hyperspectral image can be augmented into five images (including the original image) through three rotations and subsequent left–right mirroring. During the experimentation process, five-fold cross-validation is used in the calibration set, data augmentation is performed independently within each fold of training, meaning that only the data from these four folds are used for data augmentation during each four-fold training session. The large-scale hyperspectral images inputted into the MA-CNN model may occupy a significant amount of memory and pose time-consuming issues. Therefore, it is necessary to implement image normalization by scaling the pixel values to the range of 0 to 1. Subsequently, every hyperspectral image should be cropped to 224 × 224 pixels.

### 2.3. Determination of SSC in Apple Samples

The measurement of SSC in apple samples was conducted using an Abbe refractometer (Model PAL-1, manufactured by ATAGO Co., Ltd., Tokyo, Japan). The detailed operational procedure is as follows: First, mince the flesh of the apple sample, manually press it, and filter it through gauze. Then, place 2–3 drops of the juice onto the center of the prism. Once the entire prism surface is thoroughly wetted with the juice, read and record the SSC value. The average of the three measurement results for each sample is taken as the reference value for the apple’s SSC [30].

### 2.4. CNN

As a supervised deep learning approach, CNNs have made remarkable accomplishments in speech recognition, image classification, and object detection. A typical CNN architecture comprises convolutional layers, activation layers, pooling layers, fully connected layers, and batch normalization layers [31]. Serving as the core component of the CNN model, the convolutional layer achieves local connectivity and weight sharing through convolutional kernels. These kernels slide across the input feature map, performing convolution operations with the data in the receptive field to features [32]. Batch normalization (BN) is employed to mitigate internal covariate shift and expedite the training of deep neural networks. The activation function imparts nonlinear representation capabilities to the network, enhances the model’s feature representation, maps originally indistinguishable multi-dimensional features into another space, and renders the learned features more easily discernible [33]. In addition, rectified linear units (ReLU) have been used as activation functions to accelerate the convergence of neural networks. The pooling layer is usually placed after the convolutional layer to reduce the number of feature dimensions and parameters and to prevent network overfitting. Furthermore, a few fully connected layers are usually used to integrate the extracted features and generate the output through a linear activation function.

### 2.5. MA-CNN

**Figure 2:**
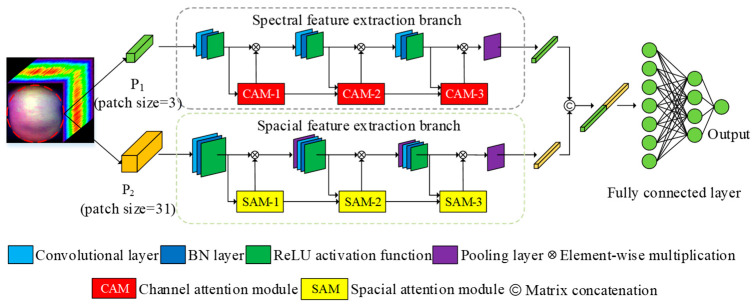
MA-CNN architecture diagram.

Figure 2 shows the overall network architecture framework, including spectral feature extraction branches based on channel attention, spatial feature extraction branches based on spatial attention, and spatial–spectral fusion feature extraction. Taking each pixel in the hyperspectral image as the center, two image blocks of different spatial sizes are constructed by taking the center pixel and its neighboring pixels. These blocks are input into the spectral feature extraction branch and the spatial feature extraction branch, respectively. Convolution, batch normalization, and pooling operations are performed to extract the spectral features of the pixel points and the spatial features composed of the center pixel and domain pixels. At the same time, shallow attention information is fused to assist the backbone network in extracting deep spectral and spatial features. Finally, deep spectral features are adaptively aggregated.

**Figure 3:**
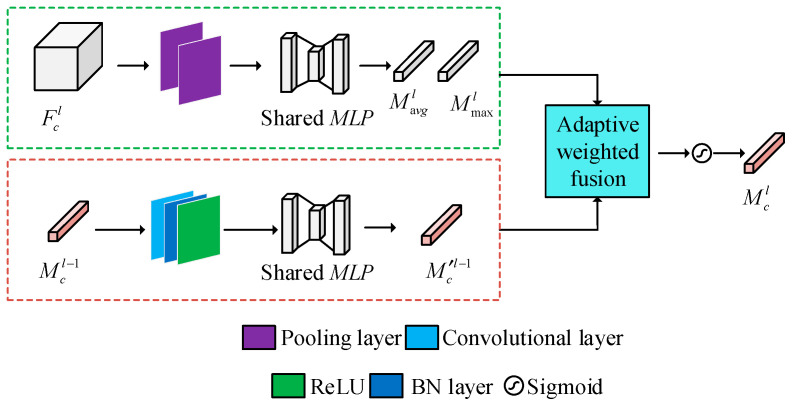
CAM module structure diagram.

Step 1: Extract spectral features based on channel attention. The structure of the channel attention module is shown in Figure 3. In the spectral feature extraction branch, 3 × 3 image blocks are selected and fed into the network. The deep spectral feature vector *F_c_* is obtained through convolution, the batch normalization layer, the ReLU activation layer, and global average pooling. The feature vector of the *l*-th layer of the spectral branch is denoted as *F^l^_c_*, which is subjected to global average pooling and regional maximum pooling along the spatial dimension to aggregate spatial features. The average pooling feature vector and maximum pooling feature vector are then passed through a multi-layer perceptron with shared weights to generate two one-dimensional feature vectors, *M^l^*_avg_ and *M^l^*_max_. The specific formula is as follows [34]:

(2) *M^l^*_avg_ = *MLP*(*AvgPool*(*F^l^_c_*))

(3) *M^l^*_max_ = *MLP*(*MaxPool*(*F^l^_c_*))

Among them, *AvgPool*(*) and *MaxPool*(*) represent the global average pooling calculation and global maximum pooling calculation along the spatial dimension, while *MLP*(*) represents a multi-layer perceptron composed of two fully connected layers and one ReLU activation function. According to Formula (4), the shallow channel attention weight *M^l^*^−1^*_c_* is introduced into the current layer features to assist in extracting deep features, and to obtain the attention weight vector *M^l^_c_* of the current layer [35].

(4) *M^l^_c_* = *σ*(*λ*_1_*M^l^*_avg_ + *λ*_2_
*M^l^*_max_ + *λ*_3_*MLP*(*W*_0_(*M^l^*^−1^*_c_*)))

*λ*_1_, *λ*_2_, and *λ*_3_ are the weight coefficients for adaptive weighted fusion in network learning, with initial values of 1.0. *W*_0_ denotes the convolutional layer that performs preliminary feature learning on the shallow channel attention vector *M^l^*^−1^*_c_*, followed by batch normalization and ReLU activation function.

Where *σ*(*) represents the sigmoid activation function, and *λ*_1_ represents the adaptive weighting of network learning. The output *M^l^_c_* is replicated through a broadcast mechanism, generating a matrix with the same dimension as *F^l^_c_* and optimizing *F^l^_c_* through element-wise multiplication [36]:

(5) *F*^′^*_c_^l^* = *F^l^_c_* ⊗ *M^l^_c_*

where ‘⊗’ denotes element-wise multiplication. Finally, the output *F_c_* of the spectral feature extraction branch is obtained through the global average pooling operation (Figure 3).

**Figure 4:**
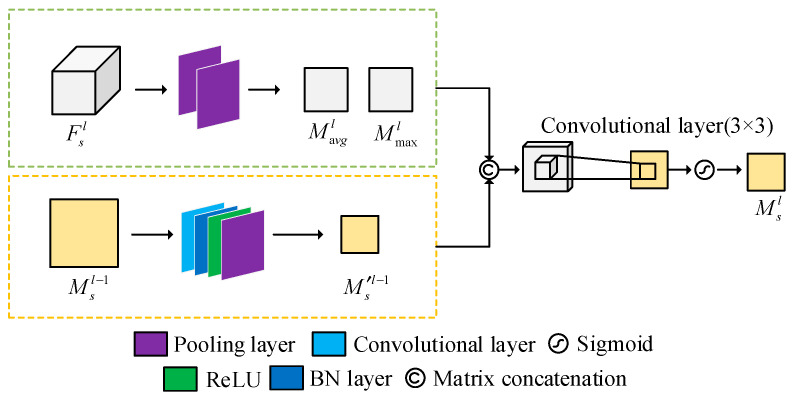
SAM module structure diagram.

Step 2: Spatial feature extraction based on spatial attention. The structure of the spatial attention module is shown in Figure 4. In the spatial feature extraction branch, image blocks within a range of 31 × 31 pixels in each hyperspectral image are taken as inputs, and deep spatial feature vectors *F_s_* are obtained through convolutional layers, batch normalization layers, ReLU activation layers, local max pooling, and global average pooling. Assuming the feature vector of the *l*-th layer of the spatial branch is *F^l^_s_*, global average pooling and global maximum pooling operations are performed along the channel dimension to aggregate spatial features, resulting in two two-dimensional feature matrices representing spatial information distribution: the average pooling feature map is represented by *M^l^*_avg_ and the maximum pooling feature map is denoted by *M^l^*_max_. The specific formula is as follows [37]:

(6) *M^l^*_avg_ = *AvgPool*(*F^l^_s_*)

(7) *M^l^*_max_ = *MaxPool*(*F^l^_s_*)

At the same time, according to Formula (8), there is a need to calculate the spatial attention weight vector, and concatenate the feature map *M^l^*^−1^*_s_* generated by the shallow (*l* − 1 layer) attention weight map *M^l^*^−1^*_s_* and the two-dimensional spatial feature maps *M^l^*_avg_ and *M^l^*_max_ generated by the current level feature map *F^l^_s_* along the channel dimension, and adaptively fuse them through a convolutional layer *W_s_* [38].

(8) *M^l^_s_* = *σ* (*W_s_*[*M^l^*_avg_, *M^l^*_max_, *W_l_* (*M*^*l*−1^*_s_*)])

Among them, *W_l_* represents the learnable weight of the convolutional layer for cross-layer transmission of spatial attention information; *W_s_* represents the convolutional layer for information fusion and spatial attention generation; and *σ*(*) represents the Sigmoid activation function. The output *M^l^_s_* is replicated along the channel dimension through a broadcast mechanism and optimized for *F^l^*_s_ through primary element multiplication [39]:

(9) *F*′*_s_^l^* = *F^l^_s_* ⊗ *M^l^_s_*

Among them, ‘⊗’ represents element-wise multiplication, the output feature *F_s_* of the spatial feature extraction branch obtained through the global average pooling operation of the main element multiplication operation (Figure 4).

Step 3: Extract spectral and spatial features. The output *F_c_* vector of the spectral feature extraction branch and the output *F_s_* vector of the spatial feature extraction branch are matrix concatenated to obtain the spatial–spectral fusion feature vector, which is then inputted into the fully connected layer to obtain the output through the activation function.

### 2.6. BOA

The BOA involves establishing a computational prior distribution based on historical evaluations of the objective function, and combining it with observation points obtained from previous iterations to obtain a posterior distribution. Subsequently, the next sample point is selected based on the posterior information, aiming to minimize the objective function. The BOA comprises two core components: the probabilistic surrogate model and the acquisition function. In this paper, Gaussian processes and the PI function are chosen as the probabilistic surrogate model and the acquisition function, respectively. In this study, the BOA is used to determine the optimal MACNN architecture and hyperparameters, including the number of kernels, activation functions, optimization algorithms, batch sizes, and learning rates for all convolutional layers.

### 2.7. Model Evaluation

The performance of the model was evaluated by the coefficient of determination (*R*^2^), root mean square error (RMSE), and residual prediction deviation (RPD). The *R*^2^ measures model accuracy, while the RMSE reflects the average difference between predicted and actual values in the corresponding set. RPD can further measure the quantitative prediction ability and stability of the model [40]. The calculation formulae for the above parameters are as follows:

(10) $$R^{2}=1-\frac{\sum_{i=1}^{N}{\left(y_{i}-{\hat{y}}_{i}\right)}^{2}}{\sum_{i=1}^{N}{\left(y_{i}-{\bar{y}}_{i}\right)}^{2}}$$

(11) $$RMSE=\sqrt{\frac{\sum_{i=1}^{N}{\left(y_{i}-{\hat{y}}_{i}\right)}^{2}}{N}}$$

(12) $$RPD=\frac{SD}{RMSE}$$

where $y_{i}$ is the reference value, ${\hat{y}}_{i}$ is the predicted value, ${\bar{y}}_{i}$ is the average of the reference values, and *n* represents the number of samples. SD stands for the standard deviation of the reference value. Generally, a well performing model should have a high *R*^2^, a low RMSE, and a high RPD. Ideally, the RPD value should be greater than 2.5 [15]. The MA-CNN is executed using a Windows 7 system, which has an Intel i7 8700 K processer with 8 cores of 3.7 GHz, and is developed based on the Keras framework (available at http://github.com/fchollet/keras, accessed on 1 November 2024), which is a Python (version 3.6) on exploiting deep learning models. In this experiment, the two equipped GPU of NIVDIA GT 1080Ti also help to improve the learning rate of MA-CNN.

## 3. Results and Discussion

### 3.1. Statistics of Reference Values

**Figure 5:**
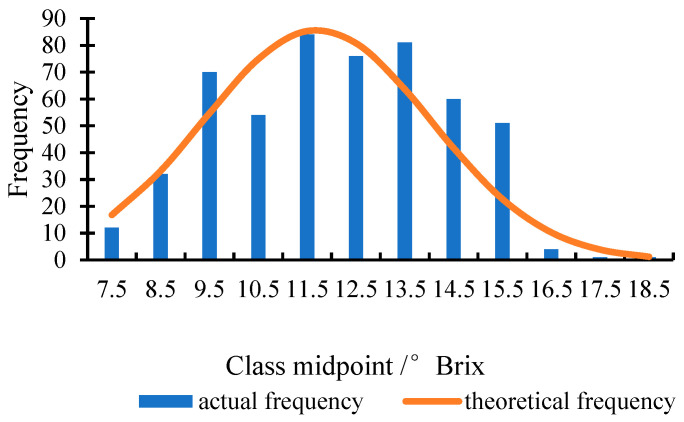
The distribution frequency histogram of SSC in the apple samples.

Table 1 shows the statistical values of SSC in apple samples. In the calibration set, the measured values of the SSC ranged from 7.20 to 18.10 °Brix, while the SSC values of the prediction set ranged from 8.05 to 15.41 °Brix. The reference range of SSC for the calibration set is broader than that of the prediction set, showing that this sample set division method is reasonable. Figure 5 illustrates the distribution frequency histogram of SSC in the apple samples. The data reveals that the data points are clustered around the mean, exhibiting an approximate normal distribution. The measured SSC values of the samples range from 7.2 °Brix to 18.10 °Brix, with an average of 11.76 °Brix and a standard deviation of 2.21 °Brix. Notably, the distribution is unimodal, with 68% of the data points clustered within one standard deviation of the mean (9.55 °Brix to 13.97 °Brix), indicating that most values are highly concentrated around the average SSC value.

### 3.2. CA-CNN Model

**Figure 6:**
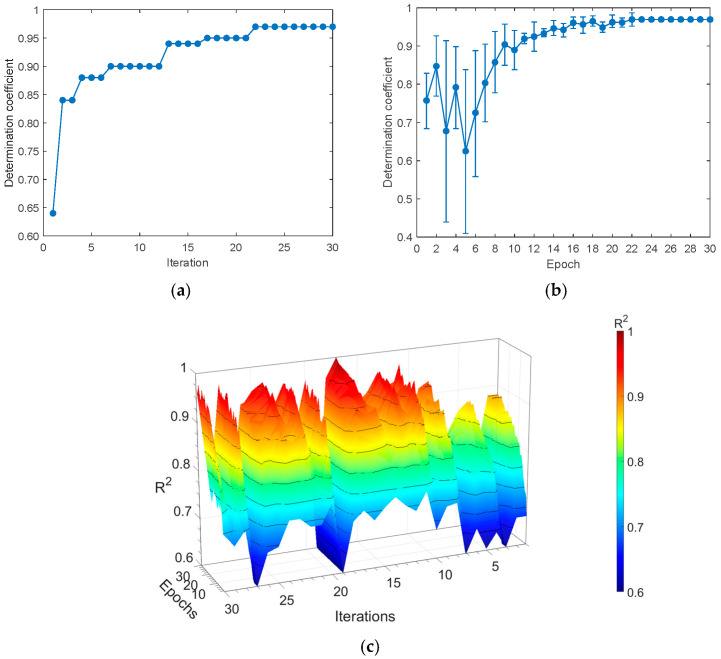
Details of the BOA as used in the CA-CNN model. (**a**) The value of *R*^2^ of the training set for each iteration; (**b**) the value of *R*^2^ of the training set for each epoch; and (**c**) the value of *R*^2^ for five-fold cross-validation.

In this study, the kernel size of the first to third convolution layers was uniformly set to three. For each max pooling layer, both the pooling size and stride were configured to two. The number of convolution kernels, activation functions, optimization algorithms, batch sizes, and learning rates for all convolution layers were optimized using the BOA. Thirty iterations were assigned to the BOA in the experiment, and the model was trained for thirty epochs. The optimization process details are illustrated in Figure 6, while the optimized parameter values for the CA-CNN model are provided in Table 2. Figure 6a indicates that the average *R*^2^ value of the training set reached its peak during the 22nd iteration, attaining a value of 0.9754. As depicted in Figure 6b, although several iterations after the 22nd iteration also achieved the highest *R*^2^ value, the 22nd iteration demonstrated superior performance stability compared to others. Consequently, CA-CNN adopted the hyperparameters from the 22nd iteration and evaluated them through five-fold cross-validation, with the corresponding performance depicted in Figure 6c. Table 2 presents the hyperparameters and their respective search spaces.

### 3.3. SA-CNN Model

**Figure 7:**
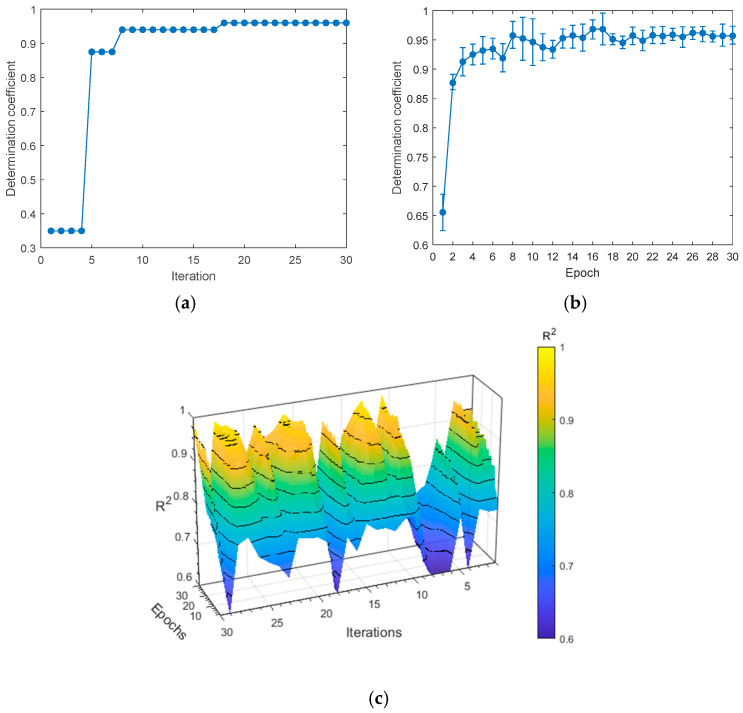
Details of the BOA as used in the SA-CNN model. (**a**) The value of *R*^2^ of the training set for each iteration; (**b**) the value of *R*^2^ of the training set for each epoch; and (**c**) the value of *R*^2^ for five-fold cross-validation.

In SA-CNN, each convolutional layer is followed by a batch normalization layer. All convolutional layers are configured with predefined kernel sizes, along with the pooling layer size and stride of the max pooling layer. Specifically, the max pooling layer has a stride of (2, 2) (2, 2) and a pooling size of (3, 3) (3, 3); the first convolutional layer employs a (3, 3) (3, 3) kernel, the second uses a (5, 5) (5, 5) kernel, and the third uses a (7, 7) (7, 7) kernel. The optimization strategy mirrors that of CA-CNN, with the training model utilizing the BOA spanning 30 iterations and 30 cycles. Figure 7a illustrates the best R^2^ value achieved in each iteration, with the highest R^2^ value occurring at the 18th iteration. Figure 7b provides a detailed depiction of the BOA iteration process. As depicted, multiple iterations after the 18th epoch attain the highest *R*^2^ value yet exhibit greater performance fluctuations compared to the 18th epoch. Consequently, the hyperparameters from the 18th iteration are ultimately selected (refer to Table 3 for specifics). Figure 7c displays the *R*^2^ value of the model under five-fold cross-validation. Table 3 enumerates the hyperparameters and their respective search spaces.

### 3.4. MA-CNN Model

**Figure 8:**
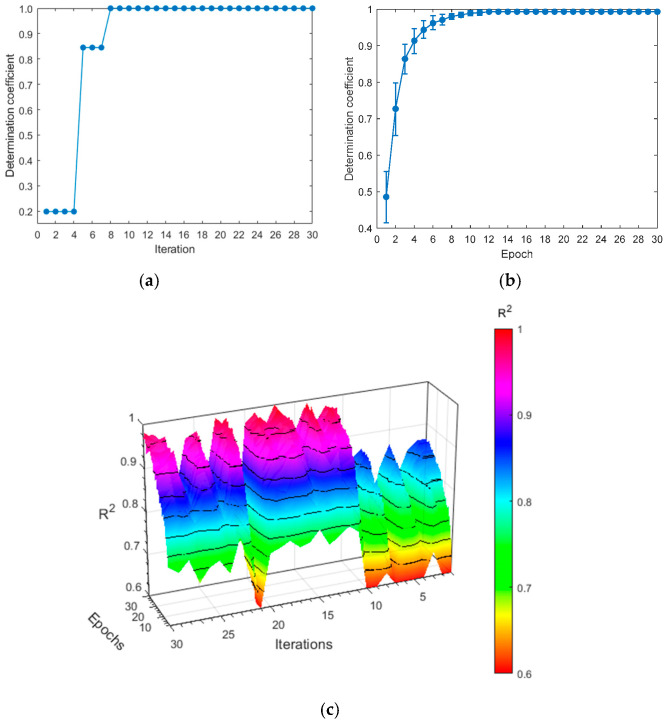
Details of the BOA, as used in the MA-CNN model. (**a**) The value of *R*^2^ of the training set for each iteration; (**b**) the value of *R*^2^ of the training set for each epoch; and (**c**) the value of *R*^2^ for five-fold cross-validation.

In the MA-CNN model, a network design with a parallel architecture is employed to segregate the spectral feature extraction, based on channel attention from the spatial feature extraction, based on spatial attention. This approach prevents mutual interference between the two processes, while reducing network depth and model complexity. The extracted spectral and spatial features are then fused and fed into a fully connected layer, with the BOA being utilized to select the model with the best performance. Figure 8 illustrates the optimization results of the SA-CNN, with the highest R^2^ value per iteration of the training set presented in Figure 8a. As illustrated in the figure, the optimal performance (0.9796) is achieved at the eighth iteration. The detailed procedure of the BOA can be found in Figure 8b. Although multiple iterations can yield optimal performance, some iterations necessitate longer training times due to an excessive number of hidden layer nodes. Consequently, the hyperparameters from the eighth iteration are ultimately chosen for their minimal number of hidden layer nodes. The *R*^2^ value of the optimized model under five-fold cross-validation is shown in Figure 8c. Table 4 provides the specific hyperparameters and their search spaces.

### 3.5. Comparison of SSC Detection Model

In this section, the model did not use a feature fusion module. The spatial and spectral features were concatenated, and output was obtained through two fully connected layers. The results are shown in Table 5, where a comparison can be made between models using the dataset of P1, P2, and the fusion data of P1 and P2. Compared to other CNN models without attention modules, the CNN model based on P2 input data exhibits the worst performance, with $R_{p}^{2}$ of 0.9389, RMSEP of 0.0906 °Brix, and RPD of 2.3529. The CNN model based on the fused data of P1 and P2 is slightly better than the CNN model based on the P1 input data, with $R_{p}^{2}$ values of 0.9578 and 0.9409, RMSEP values of 0.0743 °Brix and 0.0842 °Brix, and RPD values of 3.0635 and 2.6384, respectively.

Compared to other CNN models with attention modules, when only the spectral feature extraction branch with CA module is used, the $R_{p}^{2}$ of 0.9571, RMSEP of 0.0738 °Brix, and RPD of 2.9876, respectively. When only the spatial feature extraction branch with SA module is used, the $R_{p}^{2}$ of 0.9516, RMSEP of 0.0795 °Brix, and RPD of 2.8593, respectively. The performance in these two scenarios is close, reflecting the limitations of information utilization through single-category feature extraction, which hinder further improvement in prediction accuracy. When both branches are used together, the MA-CNN model with the fusion data of P1 and P2 obtained relatively better results, with $R_{p}^{2}$ of 0.9602, RMSEP of 0.0612 °Brix, and RPD of 3.3417, indicating that the MA-CNN model can integrate multi-layer attention information and enable the network to better focus on the feature information of key parts.

### 3.6. Comparative Evaluation and Computational Complexity Analysis of Different Models

To evaluate the performance and computational cost of the MA-CNN model, four deep learning networks were introduced: Vision Transformer (ViT) [41], Hybrid Spectral Network (HybridSN) [42], Spectral–Spatial Attention Network (SSAN) [43], and HybridViT network [44]. All models were tested using the same set of experimental samples. The MA-CNN model followed the parameter settings in this paper, while the parameters of the other models were set with reference to the relevant literature.

The experimental results are detailed in Table 6. It can be seen from Table 6 that the ViT model, due to the lack of image inductive bias built into CNN models, requires more training data to achieve the same performance. Compared to ViT, HybridSN combines the advantages of 3D convolution and 2D convolution, while SSAN introduces spatial and spectral attention mechanisms, and HybridViT integrates spatial and spectral features and combines attention mechanisms. The performance of these three methods is superior to that of ViT. A comprehensive comparison between Table 5 and Table 6 shows that the MA-CNN model, which adopts a spatial–spectral feature fusion module, optimizes the extraction process of spectral and spatial features by introducing channel attention modules and spatial attention modules in the dual-branch CNN, respectively, to generate spectral weight vectors representing the importance of spectral bands and spatial weight matrices, representing the importance of spatial neighboring pixels. This enhances key features, weakens redundant features, and effectively improves the model’s prediction performance.

To thoroughly evaluate the complexity and computational time cost of the models, this chapter tests different models using the same set of experimental samples. The computational complexity of the models is assessed by counting the number of trainable weight parameters (trainable parameters) updated during the backpropagation process of each network. Additionally, the training and testing times required by each network are recorded to quantify the computational time cost of different methods. The experimental results are presented in Table 7. The number of trainable parameters in the MA-CNN is moderate among the five comparative networks. SSAN has the lowest number of trainable weight parameters, resulting in the shortest training and testing times. Due to the quadratic growth in computational complexity and input sequence length of its self-attention mechanism, ViT has the largest number of network parameters, leading to longer model training and testing times. The MA-CNN extracts multi-dimensional features through a dual-branch architecture and adopts a spatial–spectral multi-attention strategy, which increases computational cost while improving prediction accuracy. Despite this, the method proposed in this paper is basically comparable to current mainstream algorithms in terms of training and testing time, with training time ranking third among the five methods and testing time consumption ranking fourth. More importantly, MA-CNN achieves optimal prediction accuracy within an acceptable time frame.

### 3.7. Discussion

In previous work studying the prediction of SSC in fruits, Li et al. detected SSC by using FT-NIR spectroscopy, and the results indicated that the optimal PLSR model was obtained by selecting feature bands using CARS, with a correlation coefficient of 0.92 and RMSEP of 0.661 °Brix [45]. Guo et al. used shortwave near-infrared spectroscopy to predict SSC in ‘Fuji’ apples and obtained superior performance with an R_p_ of 0.9398, and RMSEP of 0.3870% by using the ICA-SVR model [46]. These studies all employed NIR spectroscopy combined with traditional machine learning methods to establish models for predicting SSC. Near-infrared spectroscopy can only detect spectral information, and machine learning relies on traditional feature engineering, which requires extensive human experience and expert knowledge. Qi et al. utilized hyperspectral imaging combined with a convolutional neural network and a Transformer (CNN–Transformer) to analyze the SSC of cherry tomatoes, and yielded a determination coefficient of only 0.83, perhaps due to the fact that only spectral information was collected while spatial information was overlooked [47]. In contrast, the MA-CNN model proposed in this paper exhibits superior performance, benefiting from the dual-branch parallel architecture, which not only separates the extraction processes of spectral and spatial features and avoids mutual interference between the two processes, but also reduces the depth of the network and decreases model complexity.

## 4. Conclusions

In this study, the use of the MA-CNN model with the fusion data of P1 and P2 as a nondestructive method to effectively detect the SCC content in apple samples was assessed. By embedding the channel attention module and spatial attention module into the CNN, it can adaptively focus on importance and reduce redundant information interference. Compared with traditional spectral and spatial feature machine learning methods, this approach significantly improves detection performance. Subsequently, the BOA was introduced to automatically select the optimal hyperparameter combination to solve the hyperparameter optimization problem in CA-CNN, SA-CNN, and MA-CNN models. Experiments showed that the fused model was superior to CA-CNN and SA-CNN models in terms of accuracy and robustness, and the results confirmed that the combination of deep learning algorithms and hyperspectral imaging technology can effectively improve detection performance, opening up new ideas for apple quality parameter prediction and providing innovative ideas for developing fast, reliable, and nondestructive quality inspection tools for other products.

## Figures and Tables

**Table 1:** Measurement results of SSC in apple samples.

Dataset	Samples	Max (°Brix)	Min (°Brix)	Mean (°Brix)	SD (°Brix)
Calibration sets	320	18.10	7.20	12.23	2.21
Prediction sets	95	15.41	8.05	11.34	1.89
Total samples	570	18.10	7.20	11.76	1.92

**Table 2:** Optimized parameters of the CA-CNN.

Parameters	Search Space	Search Results
Number of filters of Conv1 to Conv3	(4, 64), (8, 128), (16, 256)	(32, 64, 128)
Neurons in FC1 to FC2	(32, 128), (32, 128)	(83, 51)
Learning rate	(1 × 10^−4^, 1 × 10^−1^)	0.0009
Batch size	(2, 128)	32
Activation function	[ReLU, SoftMax, Sigmoid, ELU]	ReLU
Optimization method	[SGD, Adam, AdaBound, RMSProp]	AdaBound

**Table 3:** Optimized parameters of the SA-CNN.

Parameters	Search Space	Search Results
Number of filters of Conv1 to Conv3	(4, 64), (16, 256), (8, 128)	(64, 128, 56)
Neurons in FC1 to FC2	(64, 256), (64, 256)	(125, 93)
Learning rate	(1 × 10^−4^, 1 × 10^−2^)	0.0007
Batch size	(2, 128)	53
Activation function	[ReLU, SoftMax, Sigmoid, ELU]	ELU
Optimization method	[SGD, Adam, AdaBound, RMSProp]	RMSProp

**Table 4:** Optimized parameters of the MA-CNN.

Parameters	Search Space	Search Results
Neurons in FC1 to FC2	(64, 256), (64, 256)	(86, 86)
Learning rate	(1 × 10^−5^, 1 × 10^−1^)	0.0001
Batch size	(4, 128)	69
Activation function	[ReLU, SoftMax, Sigmoid, ELU]	ReLU
Optimization method	[SGD, Adam, AdaBound, RMSProp]	AdaBound

**Table 5:** Model performance based on deep learning models constructed with different inputs.

Input Data	Model	Calibration Set		Prediction Set		
		$R_{c}^{2}$	RMSEC	$R_{p}^{2}$	RMSEP	RPD
P1	CA-CNN	0.9754	0.0698	0.9571	0.0738	2.9876
	CNN	0.9607	0.0771	0.9409	0.0842	2.6384
P2	SA-CNN	0.9732	0.0583	0.9516	0.0795	2.8593
	CNN	0.9588	0.0897	0.9389	0.0906	2.3529
Pl and P2	MA-CNN	0.9796	0.0513	0.9602	0.0612	3.3417
	CNN	0.9691	0.0659	0.9578	0.0743	3.0635

Note: P1: the image block size is 3 × 3; P2: the image block size is 31 × 31.

**Table 6:** Comparison of the performance of different models.

Model	Training Set		Test Set		
	$R_{c}^{2}$	RMSEC	$R_{p}^{2}$	RMSEP	RPD
ViT	0.9514	0.0541	0.9151	0.1496	2.7837
HybridSN	0.9633	0.0438	0.9210	0.0938	3.3121
SSAN	0.9678	0.0437	0.9230	0.0875	3.3252
HybridViT	0.9758	0.0406	0.9357	0.0806	3.2863

**Table 7:** Analysis of model complexity and computational time cost of different methods.

Methods	Trainable Params (M)	Training Time (s)	Testing Time (s)
ViT	0.25	1570	5.2
HybridSN	0.29	1457	4.8
SSAN	0.14	630	2.3
HybridViT	0.22	1289	4.1
MA-CNN	0.23	1103	4.6

## Data Availability

The original contributions presented in the study are included in the article, further inquiries can be directed to the corresponding author.
